# Multilobar massive cavitating adenocarcinoma: a case report

**DOI:** 10.1186/s13256-025-05140-2

**Published:** 2025-06-10

**Authors:** Gerardo Arwi, Paul Fogarty, Andrew Mant, Julee H’ng, Andrew Barling, Francis Thien

**Affiliations:** 1 Department of Lung and Sleep, Monash Health, 246 Clayton Rd, Clayton, VIC 3168 Australia; 2 Department of Respiratory and Sleep Medicine, Eastern Health, 8 Arnold St, Box Hill, VIC 3128 Australia; 3 Department of Oncology, Eastern Health, 8 Arnold St, Box Hill, VIC 3128 Australia; 4 Department of Pathology, Eastern Health, 8 Arnold St, Box Hill, VIC 3128 Australia; 5 Department of Thoracic Surgery, Eastern Health, 8 Arnold St, Box Hill, VIC 3128 Australia; 6 Department of Medicine, Monash University Eastern Health Clinical School, 5 Arnold St, Box Hill, VIC 3128 Australia

**Keywords:** Adenocarcinoma, Cavitary pulmonary lesion, Multilobar

## Abstract

**Background:**

The diagnostic evaluation of complex cavitary lung lesions is often challenging owing to the broad spectrum of differential diagnoses. These lesions can be associated with various conditions, making it crucial to employ comprehensive diagnostic strategies. This case underscores the significance of serial imaging, close clinical follow-up, and surgical biopsy in managing such complex cases.

**Case presentation:**

A 59-year-old woman of Indian descent was referred for the management of pneumonia. Initial chest computed tomography showed patchy inflammatory changes in the right upper lobe, a large cavity in the left upper lobe, and a smaller cavity in the left lower lobe. Follow-up imaging indicated progressive cavitary disease. Bronchoscopies did not yield significant findings. A video-assisted thoracoscopic surgery biopsy was performed, which confirmed mucinous adenocarcinoma.

**Conclusion:**

This case highlights the diagnostic challenge posed by cavitary lung lesions and emphasizes the importance of serial imaging, vigilant clinical monitoring, and surgical biopsy in achieving an accurate diagnosis. Early and systematic investigation is key to identifying rare causes such as mucinous adenocarcinoma.

## Introduction

Cavitary lung lesions carry a wide range of differentials, including infections, autoimmune diseases, and malignancies. Accurate diagnosis can often be made from radiological features and clinical context, although in complex cases tissue biopsy may be required. We report a rare case of adenocarcinoma presenting as multilobar massive cavitating disease where the diagnosis was complicated by a coexisting infection, ultimately requiring serial imaging and video-assisted thoracoscopic (VATS) biopsy for a definitive diagnosis.

## Case report

**Fig. 1:**
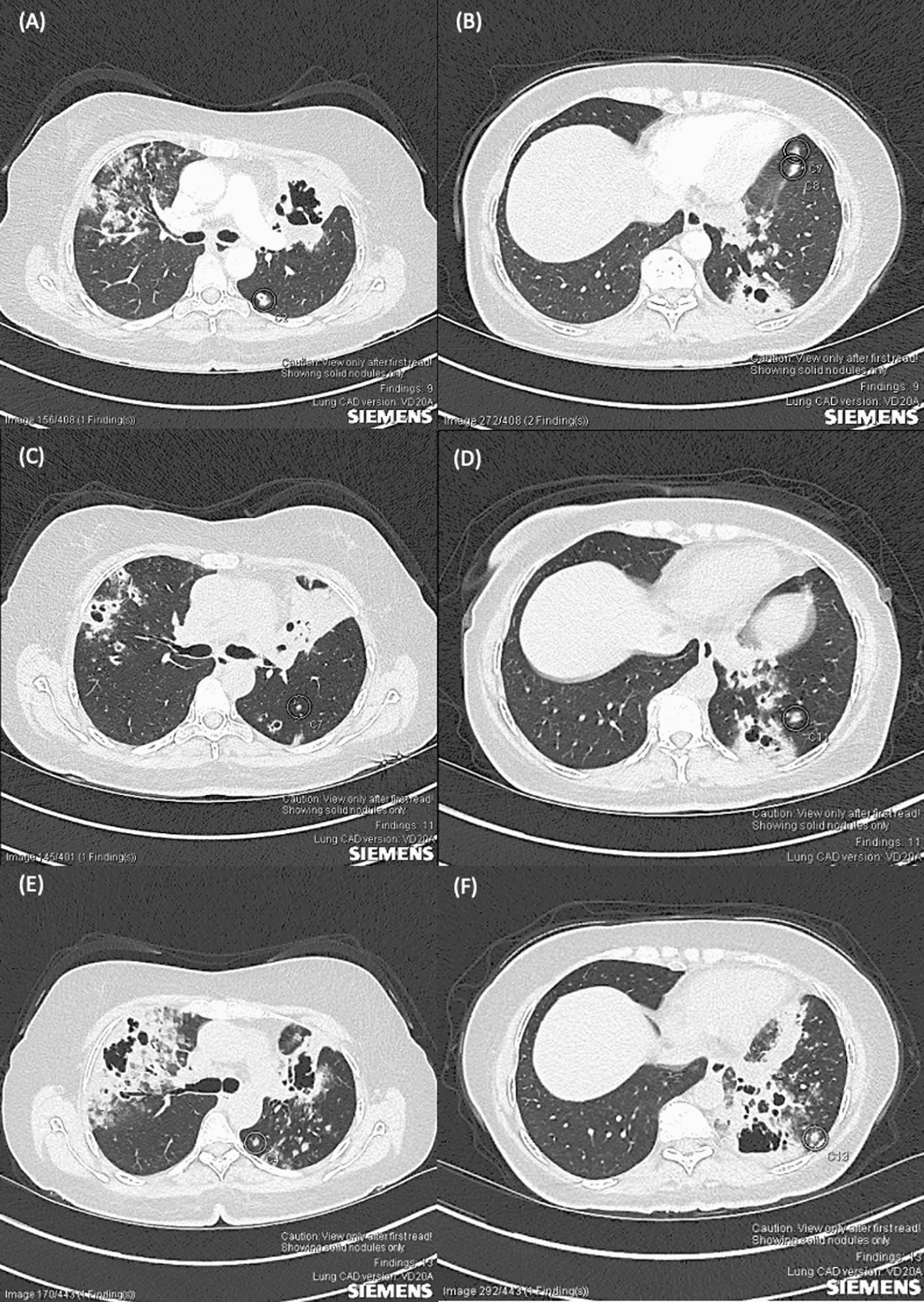
**A**, **B** Chest computed tomography pulmonary angiogram scan axial cut on admission showed extensive coarse infiltrates in the upper lobes, with extensive cavitation in the left upper lobe, and multiple small areas of cavitation bilaterally with associated fibrosis in left lower lobe; **C**, **D** imaging post-antimicrobials showed reduction in size of the left upper lobe cavitation, resolution of extensive alveolar infiltrates in right upper lobe, and no substantial changes in left lower lobe; **E**, **F** repeat imaging showed worsening cavitary lesions and new extensive centrilobular ground-glass nodules in the left upper and lower lobe

A 59-year-old woman of Indian descent was referred by her general practitioner for inpatient management of pneumonia owing to worsening cough and fever despite oral antibiotics. Her past medical history includes type 2 diabetes mellitus and hypertension. There was no family history of cancer, and she was a never-smoker. Blood test showed normal white cell count, C-reactive protein of 840 mg/L, erythrocyte sedimentation rate of 85 mm/hour, negative interferon gamma release assay, negative serum aspergillus antigen, and negative autoantibody screen. Computed tomography (CT) of the chest showed patchy inflammatory changes in the right upper lobe, with a large cavity in the left upper lobe, and a smaller cavity with associated fibrosis in the left lower lobe. (Fig. 1A, B) Transbronchial biopsy taken from the carina between left lingula and left upper lobe apicoposterior segment shows scarring and mild chronic inflammation, and bronchial washing was largely unremarkable. She improved clinically with intravenous antibiotics.

Follow-up CT imaging 2 months later showed that the left upper lobe cavity had reduced from 55 to 44 mm in diameter, and the ground-glass opacity in the right upper lobe had improved significantly. (Fig. 1C, D) Improvement in her left upper lobe suggested an infective process for her presentation, and she was commenced on a period of oral antibiotics to ensure resolution.

**Fig. 2:**
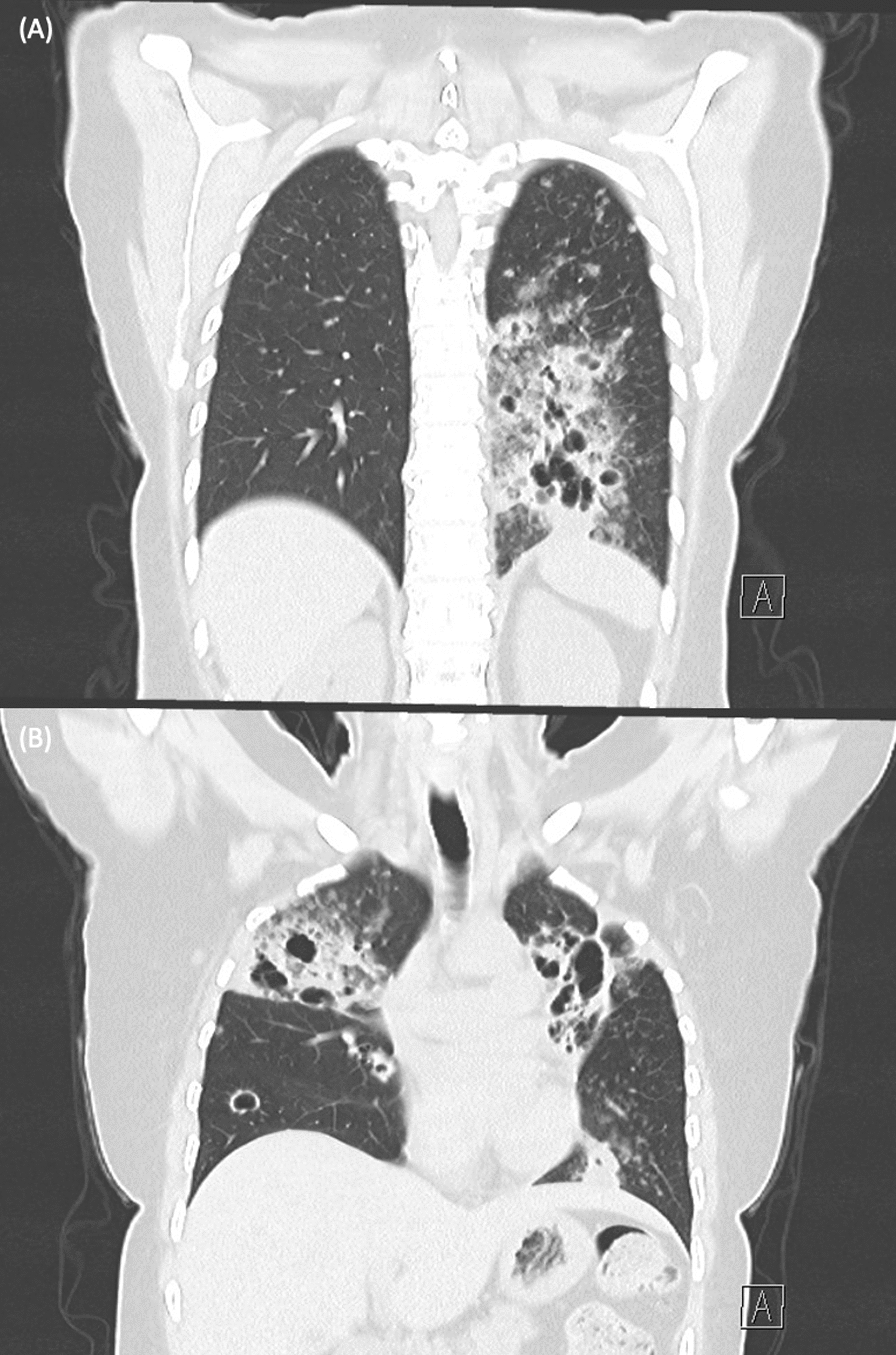
**A**, **B** Coronal view of computed tomography imaging showing multilobar cavitary lesions and extensive centrilobular ground-glass nodules

**Fig. 3:**
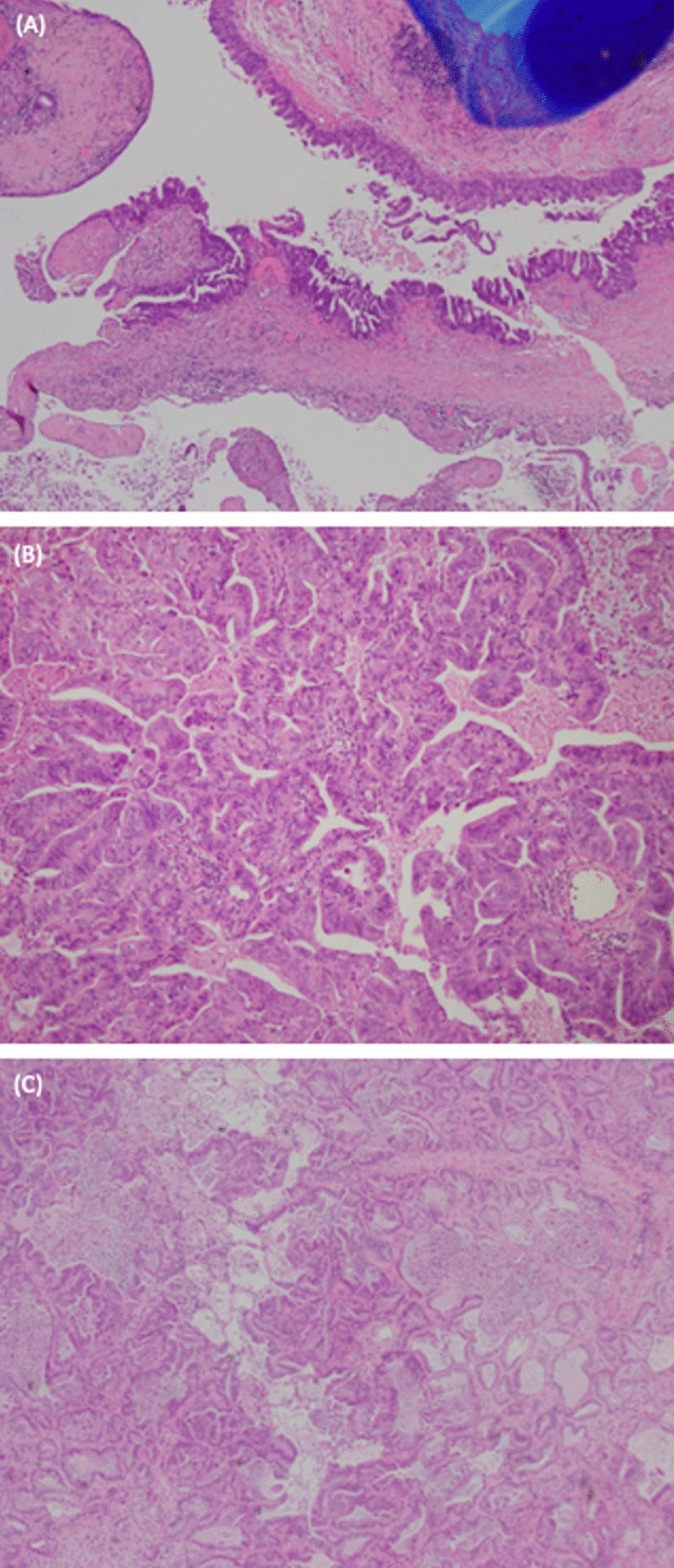
H&E-stained section revealing moderately differentiated mucinous adenocarcinoma with invasive components showing acinar and papillary architectures; (**A**, **B**) left upper lobe (**C**) left lower lobe

She remained clinically stable throughout subsequent follow-ups. However, repeat CT imaging 4 months later showed progressive cavitary disease in right upper lobe, left upper lobe, and left lower lobe. (Figs. 1E, F and 2A, B). Blood tests, including inflammatory markers and autoantibodies were unremarkable, as were repeat transbronchial biopsy and bronchial washings. Triple-port VATS wedge resections of the left lingula and left lower lobe were performed for definitive diagnosis, and the biopsy revealed mucinous adenocarcinoma. (Fig. 3) Hence, the differentials for these lesions include three synchronous lung primaries, at least one lung primary with metastases, or multiple foci of metastatic adenocarcinoma from another site. She was subsequently referred to medical oncology for further management.

## Discussion

We report a rare case of adenocarcinoma presenting as massive multilobar cavitation, which was complicated by co-existent bacterial infection and improved with antibiotics. Multiple flexible bronchoscopies did not reveal evidence of malignancy, and only after multiple reviews and VATS biopsy was the final diagnosis of adenocarcinoma made.

A lung cavity is defined as a gas-filled space within pulmonary consolidation, mass, or nodule [1]. Differential diagnoses of cavitary lung lesions include infectious processes such as necrotizing infections or septic emboli; or noninfectious diseases, such as vasculitis, sarcoidosis, or malignancies [2]. Cavitations occur in 22% of primary lung cancer, with squamous cell carcinoma being the most common, followed by adenocarcinoma and large cell carcinoma [3, 4]. In contrast, only 4% of lung metastases cavitate [5]. The mechanism of cavity formation in primary lung cancer may be owing to rapid tumor growth exceeding vascular supply resulting in central necrosis. One study reported that 81% of tumors with cavitation over-expressed epidermal growth factor receptor, which may lead to rapid growth, resulting in central necrosis and cavity formation [4]. Radiological features of cavitations suggesting malignancy include wall thickness, spiculation, or irregular margins [6, 7].

Pulmonary cavitation is the classic hallmark of *Mycobacterium tuberculosis* [8]. Cavities create an immune-sheltered zone for bacterial growth owing to poor phagocyte and granulocyte penetration into the necrotic center [9]. Consequently, cavitation is indicative of high bacillary burden, with bacterial loads of up to 10^7^−10^9^ bacilli/gram [9]. The presence of cavities in tuberculosis is associated with poorer outcome, higher transmission rate, and increased risk of treatment failure [10]. Other CT findings suggestive of active tuberculosis include lymphadenopathy with peripheral rim enhancement and central low attenuation, dense and homogeneous consolidation, and pleural effusions [10, 11]. Fungal infections can cause invasive cavitating lung lesions, especially in those immunocompromised with chronic respiratory diseases [12]. CT findings suggestive of invasive fungal disease include scattered nodules, masses, or patchy foci, as well as micronodules surrounded by a circular area of ground-glass opacity known as the halo sign [13]. Granulomatosis with polyangiitis (GPA) is another differential consideration, [14] with pulmonary nodules being the most common lung manifestation and cavitation in approximately 25% of nodules being greater than 2 cm [15]. Other radiological manifestations for GPA include consolidation, ground-glass changes due to alveolar hemorrhage, mosaic attenuation, and tree-in-bud manifestations attributed to arteriolar involvement [16].

Navigation bronchoscopy studies demonstrate a diagnostic yield of around 71% when used to sample pulmonary nodules [17]. Advanced methodologies, including robot-assisted bronchoscopy and tomosynthesis-based fluoroscopic navigation, can enhance diagnostic yield. Factors that increase the success of bronchoscopy include positive bronchus sign and nodules measuring ≥ 20 mm [17]. Navigation bronchoscopy has an excellent safety profile, with the prevailing adverse events being pneumothorax (2.5%), bleeding (2.1%), and infection (0.2%) [17]. Failure to diagnose adenocarcinoma in this case may be attributed to distance of cavitations from the airway and sampling error.

Two other cases of multiple cavitary lung adenocarcinoma have been reported. (Table 1) Unique features of our case include the large dimensions of multilobar cavitations and coexisting infection, which complicated diagnosis. This report highlights the importance of serial imaging, close follow-up, and the role of surgical biopsy for definitive diagnosis of complex cavitary lesions.

**Table 1:** Clinical presentation and management of all available reports of patients with multilobar massive cavitating adenocarcinoma

Patient	First author, journal	Year; volume: page	Age	Sex	CT finding	Diagnostic modality	Biopsy findings	Treatment
1	Nakamura S. Cureus	2021; 13: e13795	78	Female	Multiple pulmonary nodules and cavities, thickening of interlobular septa and bronchovascular bundles and bilateral pleural effusions	Postmortem autopsy	Columnar malignant cells containing abundant mucin, invading into lymphatic vessels	Palliative
2	Carreto L, Arch Bronconeumol	2019; 55: 495–496	62	Female	Multiple bilateral thin-walled air-filled cavities, with diffuse distribution but basal predominance, round shaped but some with irregular borders, and centrilobular nodules	Surgical lung biopsy	N/A	Chemotherapy

## Conclusion

- Adenocarcinoma can manifest as massive multilobar cavitation complicated by co-existent bacterial infection.
- This report highlights the importance of serial imaging, close follow-up, and the role of surgical biopsy for definitive diagnosis of complex cavitary lesions.

## Data Availability

Not applicable.
